# Hematocrit value at early middle age predicts hypertension at late middle age; the Tampere adult population cardiovascular risk study, a 30-year follow-up

**DOI:** 10.1016/j.pmedr.2023.102192

**Published:** 2023-03-31

**Authors:** Jaakko Piesanen, Tarja Kunnas, Seppo T Nikkari

**Affiliations:** Department of Medical Biochemistry, Faculty of Medicine and Health Technology, Tampere University, Tampere FI-33014, Finland

**Keywords:** Hematocrit, Vascular disease, Death

## Abstract

A 50-year-old cohort from the Tampere adult population cardiovascular risk study having hypertension and their controls were examined retrospectively at the age of 35 years, and followed up prospectively up to the age of 65 years to determine whether an early hematocrit (HCR) measurement predicts later hypertension or cardiovascular complications. A total of 307 subjects having hypertension and 579 non-hypertensive controls were chosen from the 50-year-old cohort and regrouped according to HCR values obtained when they were 35 years old, one with HCT < 45 % (n = 581), and the other, with HCT ≥ 45 % (n = 305). Hypertension and coronary artery disease (CAD) by the age of 60 years were determined by self-report and the National Hospital Discharge Registry. Outcomes for death up to the age of 65 years were collected from the National Statistics Centre. HCT ≥ 45 % at the age of 35 years associated with hypertension (p = 0.041) and CAD (P = 0.047) by the age of 60 years. When the subjects were followed up to the age of 65 years, HCT ≥ 45 % associated with premature cardiovascular death (P = 0.029), and death by any cause (P = 0.004). These results were obtained after adjusting for BMI-class recorded at 50 years of age. However, when outcome was also adjusted by gender, current smoking, vocational education, and state of one's health, association of the ≥ 45 % group with CAD and death was abolished. The association with hypertension remained (P = 0.007). In conclusion, there was a significant association of HCT ≥ 45 % at early middle age with subsequent hypertension.

## Highlights

• Hematocrit values of ≥ 45 % at early middle age predicted later hypertension.
• Hematocrit ≥ 45 % also predicted later coronary artery disease and mortality.
• After adjusting for gender, only the strong association with hypertension remained.
• Nearly all subjects in the hematocrit ≥ 45 % group were men.

## 1 Introduction

It has been shown that hematocrit (HCT) levels in men are associated with the incidence of hypertension independent of other risk factors (Jae et al. 2014). We have previously shown that HCT values of ≥ 50 % observed in a cohort of 55-year-old men were associated with increased coronary heart disease (CHD) mortality during a subsequent 28-year follow-up (Kunnas et al. 2009). Men with HCT ≥ 50 % were 2.4 times more likely to die from CHD than were men with HCT < 50 %, proposing that for men over 55 years of age such HCT levels might be an additional risk factor (Kunnas et al. 2009). Others have reported that in patients with polycythemia vera, those with a HCT of<45 % had a significantly lower rate of cardiovascular death and major thrombosis than did those with HCT of 45 % to 50 % (Marchioli et al. 2013).The results of other studies of patients with primary proliferative polycythemia have also indicated that optimum HCT level was lower than is often assumed and should be maintained at<45 % to decrease the risk of vascular occlusive episodes (Pearson and Wetherley-Mein, 1978, Messinezy et al., 1985). The present study examined whether HCT ≥ 45 % determined at early middle age (35 years) predicts hypertension and coronary artery disease (CAD) at late middle age (60 years), and mortality at late adulthood (65 years).

## 2 Materials and methods

### 2.1 Subjects

The Tampere adult population cardiovascular risk (TAMRISK) study data used in the present study was collected from periodic health examinations (PHE) in 2003–2006 for 50-year-old women and men in Tampere, a city in southern Finland with 220 000 inhabitants (Määttä et al., 2015). The subjects chosen for this study also had PHE data from 1988 to 1990 when they were 35 years old. Health and past medical history were assessed using a structured questionnaire. Information on socioeconomic factors, such as vocational education (scale1-5), and other health/chronic conditions, such as state of one's health (scale 1–5) were also recorded on the 50-year visit. Body mass index (BMI) was calculated from height (cm) and weight (kg), and the subjects were divided to BMI classes of < 25, 25 to 29.9, 30 to 34.9, and ≥ 35 kg/m^2^. In 1988–1990 HCT (percent) was analyzed by the spun microhematocrit method. Cases were subjects who had reported hypertension at the age of 50 years (n = 307, 38 % women) (as diagnosed by a physician) and for each case, at least one normotensive control subject with the same sex, and similar smoking habits, was chosen (n = 579, 39 % women). All participants gave informed consent, and the Ethics Committee of the Tampere University Hospital approved the study. The stages of adult life referred to are early middle age (ages 35--44), late middle age (ages 45--64), and late adulthood (ages 65 and older) (Medley. 1980).

### 2.2 Outcomes

For retrospective and prospective follow-up, the original cases and controls were combined. Using the patient's national identity code, data on hospitalizations including ICD‐10 codes for discharge diagnoses were obtained from the National Hospital Discharge Registry (HILMO) maintained by the National Institute of Health and Welfare. Prevalence of ischemic heart diseases (CAD) (I20‐I25) were followed up to 2014 until the subjects were on the average 60 years old (58–61 years). In follow‐up of the subjects, there were 70 with CAD (13 women, 57 men). Vital status, allowing a maximum age of 65 years for participants who had been under follow-up for the longest time, was ascertained based on social security number and the cause of death from death certificates. This information was obtained up to December 2018 from the National Statistics Centre (Statistics Finland). ICD10 codes were used to group cause specific mortality for cardiovascular disease (0 women, 12 men) (ICD10: I21, I25, I71), and all causes of death which included cardiovascular disease, cancer, respiratory disease, accidents, suicide, and violence (3 women, 32 men).

### 2.3 Statistical analysis

Chi-square test or Fisher’s exact test for categorical variables or logistic regression were applied for the comparison of groups. Kaplan-Meier survival analysis for cardiovascular deaths was also performed. Analyses were carried out using SPSS 23.0 for Windows (SPSS Inc., Chicago, Illinois, USA).

## 3 Results

Background characteristics of the hypertensive case group and their controls without hypertension at the age of 50 years have been previously described (Maatta et al. 2013).

The overall range of HCT values at the age of 35 years was from 31 % to 53 %. HCT was higher in men (45 % ± 2.7 %) than in women (40 % ± 2.4 %). The subjects were divided into two groups, one with HCT < 45 % (n = 581), and the other, HCT ≥ 45 % (n = 305). Clinical characteristics of these groups at the age of 35 years are shown in Table 1. Nearly all subjects in the HCT ≥ 45 % group were men (98 %). This group also had higher BMI and blood pressure compared to the HCT < 45 % group.

**Table 1:** Clinical characteristics of the study population stratified according to HCT < 45 % and HCT ≥ 45 % at the age of 35 years.

HCT-class (n)	HCT < 45 % (5 8 1)	HCT ≥ 45 % (3 0 5)	P-value*
HCT (SD)	41 % (2 %)	47 % (2 %)	<0.001
Body mass index, kg/m^2^ (SD)	24.2 (3.7)	25.4 (3.8)	<0.001
Male gender, %	42	98	<0.001
Systolic blood pressure, mmHg (SD)	126.0 (12.2)	132.0 (11.5)	<0.001
Diastolic blood pressure, mmHg (SD)	79.5 (9.6)	84.9 (7.9)	<0.001

SD, standard deviation. *T-test or chi-square test.

By the age of 60 years, HCT ≥ 45 % at the age of 35 years associated with hypertension (P = 0.003) and CAD (P = 0.022), even after adjusting for BMI-class at 50 years of age (P = 0.041 and P = 0.047, respectively) (Table 2). When the subjects were followed up to the age of 65 years, after adjusting for BMI-class, HCT ≥ 45 % was associated with premature cardiovascular death (P = 0.029), and risk of death by any cause (P = 0.004).

**Table 2:** Outcome of the study population stratified according to HCT < 45 % and HCT ≥ 45 % at the age of 35 years.

HCT-class (n)	HCT < 45 % (5 8 1)	HCT ≥ 45 % (3 0 5)	P*	P**	OR**	95 % CI**	P***	OR***	95 % CI***
Hypertension by the age of 60 years % (n)	31.2 (1 8 1)	41.3 (1 2 6)	**0.003**	**0.041**	1.4	1.0–1.9	**0.007**	1.7	1.2–2.5
CAD by the age of 60 years % (n)	6.4 (37)	10.8 (33)	**0.022**	**0.047**	1.7	1.0–2.7	0.912	1.0	0.6–2.0
Cardiovascular death by the age of 65 years % (n)	0.7 (4)	2.6 (8)	**0.029**	**0.029**	3.9	1.2–13.0	0.477	1.6	0.5–5.4
Death by any cause by the age of 65 years % (n)	2.4 (14)	6.9 (21)	**0.002**	**0.004**	2.8	1.4–5.6	0.347	1.4	0.7–3.1

CAD, coronary artery disease. * Chi-square or Fisher’s exact test. ** Logistic regression adjusted by BMI-class, recorded at the age of 50 years. *** Logistic regression adjusted by gender, current smoking, BMI-class, vocational education, and state of one's health, recorded at the age of 50 years. P values < 0.05 are in bold.

**Fig. 1:**
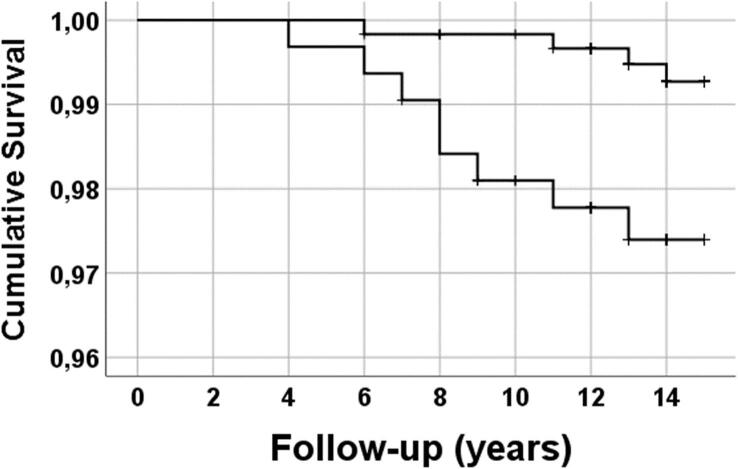
Kaplan-Meier survival analysis from cardiovascular deaths of subjects with HCT < 45 % (upper curve) and those with HCT ≥ 45 % (lower curve) (P = 0.015). The subjects were followed from 50 up to 65 years of age.

In follow-up of the subjects from 50 up to 65 years of age, the Kaplan-Meier survival curve (Fig. 1) illustrates the better survival from cardiovascular death of subjects with HCT < 45 % (upper curve) compared to those with HCT ≥ 45 % (lower curve) (P = 0.015).

However, when outcome of the study population was adjusted by gender, current smoking, BMI-class, vocational education, and state of one's health, recorded at 50 years of age, association of the ≥ 45 % group with CAD and death was abolished (Table 2). The association with hypertension remained (OR 1.7, 95 % CI 1.2–2.5, P = 0.007).

## 4 Discussion

HCT is the volume percentage red blood cells in blood. An elevated HCT may signify polycythemia, characterized by thrombotic predisposition, possibly by hyperviscosity of blood. The threshold to diagnosis of polycythemia by the World Health Organization (WHO) classification is HCT value of 49 % for men, and 48 % for women, respectively (Iurlo et al. 2020). We have previously shown in a cohort of 55-year-old men of the TAMRISK study that HCT values of ≥ 50 % were associated with increased CHD mortality during a subsequent 28-year follow-up (Kunnas et al. 2009). There are also several previous studies that have reported an association of HCT with CHD (Gagnon et al., 1994, Carter et al., 1983, Sorlie et al., 1981, Erikssen et al., 1993). In these studies, the follow-up was started at late middle age (ages 45–64 years). However, the present study is the first to demonstrate an association of HCT values of 45 % or over at early middle age (age of 35 years) with subsequent hypertension.

We report that HCT of ≥ 45 % was associated with CAD, premature cardiovascular death, and risk of death by any cause, when outcome was only adjusted by BMI-class. However, when outcome was adjusted by gender, current smoking, BMI-class, vocational education, and state of one's health, recorded at 50 years of age, association of the ≥ 45 % group with CAD and death was abolished. This was because there were only seven women in the HCT ≥ 45 % group of whom two had outcome of hypertension, but none of them had CAD or had died. Men have higher reference range of HCT (39 %-50 %) compared to women (35 %-46 %). It has been speculated that in women, the lower HCT may result in reduced plasma viscosity and a lower risk for CAD compared to men (Paul et al. 2012). Thus, reference values may not mean that they are the ones that one would recommend, especially for men. It has previously been shown in hypertensive patients that in absolute HCT values, HCT > 44 % in both men and women is associated with increased risk of death (Paul et al. 2012). There may be an advantage in the lower HCT levels in women, who live longer and have lower CAD risk than men. Whether lowering HCT levels in men would have a positive effect on cardiovascular risk remains to be seen (Murphy. 2014).

Elevated blood viscosity has been thought to explain the harmful effects of elevated HCT levels on cardiovascular health, but whether HCT levels independently increase the risk of cardiovascular disease is still unclear. HCT level itself has been found to be affected by risk factors for cardiovascular diseases, such as smoking, obesity, poor sleep (Yen Jean et al., 2019), and metabolic syndrome (Huang et al., 2018, Zeng et al., 2015, Tamariz et al., 2008).

One explanation for the gender difference in HCT may be the higher level of serum testosterone in men compared to women. Testosterone suppresses hepcidin, which is an inhibitor of iron absorption from the gut and reticuloendothelial cells into the circulation. When hepcidin is suppressed, serum iron overload promotes erythrocytosis, which in turn leads to increase in HCT ([Bibr b0010][Bibr b0005], Hennigar et al., 2020). Proteins encoded by the genes *HFE, HJV,* and *BMP4* participate in signaling routes controlling the production of hepcidin. Associations between genetic variants in these genes and arterial hypertension have been shown earlier (Ellervik et al., 2010, Määttä et al., 2015, Nikkari et al., 2017; Piesanen et al., 2017; Selvaraj et al., 2019). Whether there is another effect of hepcidin on hypertension than through HCT remains to be shown.

The limitations off the study include a relatively small population of ethnic Finns, and the results may not be generalized to other ethnic populations. Moreover, the population with HCR ≥ 45 % consisted almost totally of men. Only seven women in the group reduces the validity and generalizability of our conclusions specifically in relation to the female population. Other limitations include possible residual confounding because limited background data was available at baseline.

### 4.1 Conclusion

We found that there was a significant association of HCT ≥ 45 % at early middle age with subsequent hypertension. Our finding underlines the potential role of HCT as a predictive marker for hypertension, and HCT levels of<45 % seem to be beneficial in the general population. These observations are in line with the findings that a HCT target of<45 % in patients with polycythemia is associated with a significantly lower rate of thrombotic complications, as compared with a target of 45 % to 50 % (Marchioli et al., 2013, Pearson and Wetherley-Mein, 1978, Messinezy et al., 1985). A larger population and varying ethnic groups could be a springboard for finding a common HCT target for both women and men.

## CRediT authorship contribution statement

**Jaakko Piesanen:** Writing – review & editing. **Tarja Kunnas:** Investigation, Writing – review & editing. **Seppo T Nikkari:** Formal analysis, Writing – original draft, Writing – review & editing.

## Declaration of Competing Interest

The authors declare that they have no known competing financial interests or personal relationships that could have appeared to influence the work reported in this paper.

## Data Availability

Data obtained from the National Hospital Discharge Registry (HILMO) are confidential under the Act on National Personal Data Registers kept under the Finnish Health Care System.
